# NGLY1 Deficiency: A Rare Newly Described Condition with a Typical Presentation

**DOI:** 10.3390/life11030187

**Published:** 2021-02-27

**Authors:** Ivana Dabaj, Bénédicte Sudrié-Arnaud, François Lecoquierre, Kimiyo Raymond, Franklin Ducatez, Anne-Marie Guerrot, Sarah Snanoudj, Sophie Coutant, Pascale Saugier-Veber, Stéphane Marret, Gaël Nicolas, Abdellah Tebani, Soumeya Bekri

**Affiliations:** 1 Department of Neonatal Pediatrics, Intensive Care and Neuropediatrics, Normandie University, UNIROUEN, CHU Rouen, INSERM U1245, 76000 Rouen, France; ivana.dabaj@chu-rouen.fr (I.D.); franklin.ducatez@gmail.com (F.D.); stephane.marret@chu-rouen.fr (S.M.); 2 Department of Metabolic Biochemistry, Normandie University, UNIROUEN, CHU Rouen, INSERM U1245, 76000 Rouen, France; b.sudrie-Arnaud@chu-rouen.fr (B.S.-A.); sarah.snanoudj@chu-rouen.fr (S.S.); abdellah.tebani@chu-rouen.fr (A.T.); 3 Department of Genetics and Reference Center for Developmental Disorders, FHU G4 Génomique, Normandie University, UNIROUEN, CHU Rouen, INSERM U1245, 76000 Rouen, France; francois.lecoquierre@chu-rouen.fr (F.L.); anne-marie.guerrot@chu-rouen.fr (A.-M.G.); sophie.coutant@inserm.fr (S.C.); pascale.saugier-veber@chu-rouen.fr (P.S.-V.); gael.nicolas@chu-rouen.fr (G.N.); 4 Department of Laboratory Medicine and Pathology, Mayo Clinic, Rochester, MN 55902, USA; kimiyo.raymond@mayo.edu

**Keywords:** NGLY1-CDDG, NGLY1, congenital disorder of deglycosylation, hypolacrimia, alacrimia, movement disorder, developmental delay, hypotonia, elevated transaminases

## Abstract

NGLY1 deficiency is the first recognized autosomal recessive disorder of N-linked deglycosylation (NGLY1-CDDG). This severe multisystemic disease is still poorly known and, to date, most cases have been diagnosed through whole exome or genome sequencing. The aim of this study is to provide the clinical, biochemical and molecular description of the first NGLY1-CDDG patient from France along with a literature review. The index case presented with developmental delay, acquired microcephaly, hypotonia, alacrimia, feeding difficulty, and dysmorphic features. Given the complex clinical picture and the multisystemic involvement, a trio-based exome sequencing was conducted and urine oligosaccharides were assessed using mass spectrometry. The exome sequencing revealed a novel variant in the *NGLY1* gene in a homozygous state. NGLY1 deficiency was confirmed by the identification of the Neu5Ac1Hex1GlcNAc1-Asn oligosaccharide in the urine of the patient. Literature review revealed the association of some key clinical and biological features such as global developmental delay—hypertransaminasemia, movement disorders, feeding difficulties and alacrima/hypolacrima.

## 1. Introduction

NGLY1 deficiency (OMIM#615273) is a rare autosomal recessive congenital disorder of N-linked deglycosylation (NGLY1-CDDG) related to N-Glycanase (EC 3.5.1.52) deficiency. This disease is the only congenital disorder of deglycosylation while more than 100 different congenital disorders have been reported for glycosylation [1]. The N-Glycanase enzyme and the corresponding gene, *NGLY1*, have been characterized for several decades [2], while the first description of NGLY1 deficiency in humans was reported by Need et al. in 2012 [3]. To date, 36 cases with a confirmed molecular diagnosis have been described while the NGLY1 Foundation, a patient support and research organization for NGLY1 Deficiency (NGLY1.org), reported in 2018 sixty-three patients aged from a few months to 22 years with different ethnic backgrounds [1,3,4,5,6,7,8,9,10,11]. The cardinal clinical signs are as follows: developmental delay, hypolacrimia or alacrimia, hypotonia, elevated transaminases and acquired microcephaly. 

**Figure 1:**
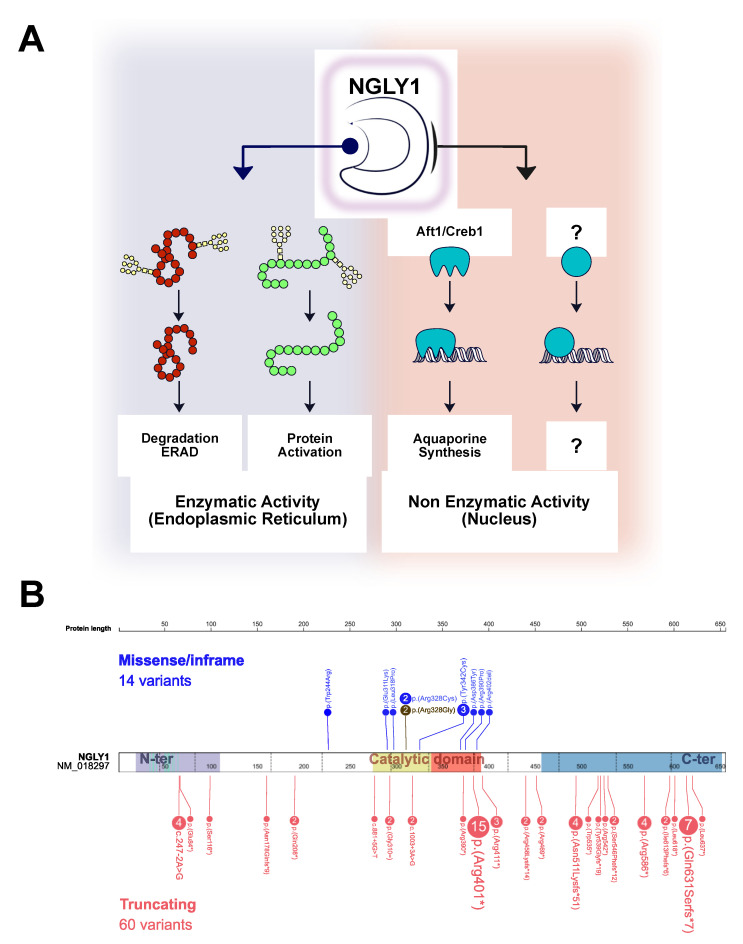
(**A**). Enzymatic and non-enzymatic activities of N-glycanase. ERAD: Endoplasmic Reticulum Associated Degradation, N-ter: N-terminal, C-ter: C-terminal. (**B**). Molecular variations of thirty-seven described cases including that of the present case (highlighted in a colored background). The number of mutant alleles is indicated between brackets. C-ter: C-terminal domain; N-ter: N-terminal domain.

During the N-linked protein glycosylation process, the oligosaccharide (glycan) is linked to selected asparagine residues of polypeptides through its proximal N-acetylglucosamine. The N-Glycanase cleaves the N-glycan from the asparagine residue and thus catalyzes the removal of N-linked oligosaccharides from misfolded glycoproteins prior to their degradation through the endoplasmic reticulum-associated degradation (ERAD) process. The NGLY1 structure reveals three domains: N-terminal ubiquitin-binding domain, catalytic domain and C-terminal mannose-binding domain [12]. The pathophysiology basis of NGLY1 deficiency may be related to the accumulation of misfolded glycoproteins [7]. In addition, it has recently been shown that this catalytic function of deglycosylation is involved in the activation of proteins such as the transcription factor Nuclear Factor Erythroid 2 like 1 (NFE2L1) that regulates the proteasome, mitochondria and inflammation processes [13,14,15]. Besides, several non-enzymatic functions have been attributed to NGLY1 and may contribute to the large spectrum of clinical symptoms associated with NGLY1 deficiency. Thus, NGLY1 regulates the transcription of water channel aquaporins independently from its catalytic activity. Aquaporins play a major role in tear secretion and brain functions. The decrease of aquaporin expression may underlie the hypolacrimia/alacrimia and neurological disturbances observed in NGLY1 deficiency (Figure 1A) [16]. These numerous roles of NGLY1 are certainly at the origin of the multisystemic involvement in this complex disease. Regarding the treatment, there is no FDA-approved treatments for NGLY1-CDDG. There are preclinical studies on enzyme replacement therapy and screens for endo-β-N-acetylglucosaminidase inhibitors which could be potentially used to treat NGLY1 deficiency [17,18]. Here, we report the first case from France and provide a literature review.

## 2. Patients and Methods

### 2.1. Case Description

**Figure 2:**
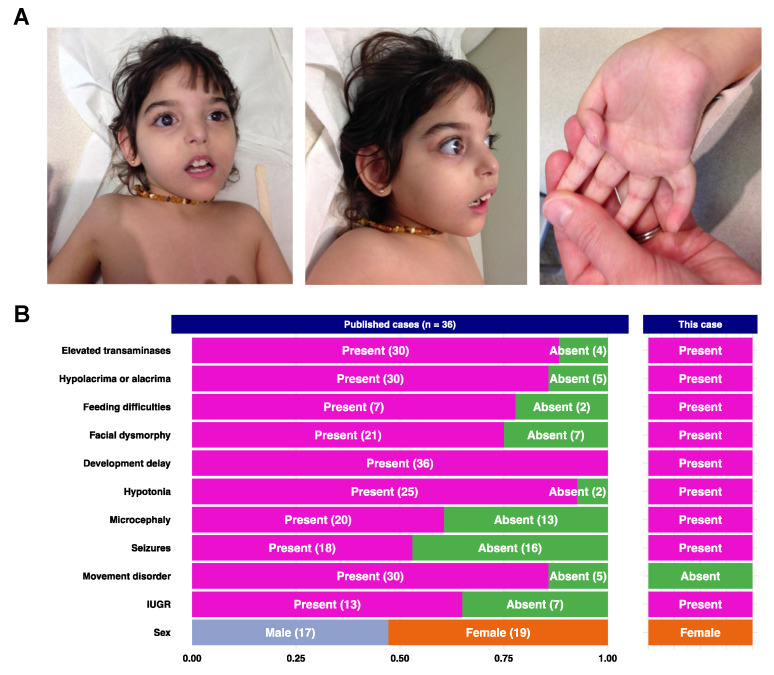
(**A**). Photo of the index case presenting dysmorphic features: (left, middle) facial dysmorphism: small mouth, long palpebral fissure, hypertelorism, low-set ears, high-arched palate, corneal opacity. (right) hand abnormality: clasped thumb, clinodactyly. (**B**). Phenotype summary of the current case and previously reported cases [1,3,4,5,6,7,8,9,10,11,23,25,26,27,28]

We describe the case of a girl who was first presented to our department at the age of 3 years with developmental delay, microcephaly, hypotonia, alacrimia, feeding difficulties, epilepsy, hypertransaminasemia, recurrent inhalation pneumonia. The patient was born at 33 weeks of gestation, from consanguineous parents, birth weight 1760g (37th percentile), height 44 cm (64th percentile), head circumference 31 cm (< 2nd percentile), with APGAR scores of 9 and 10 at 1 and 5 min, respectively. There was a history of death in the neonatal period of a brother and a cousin, of unknown etiology. She was hospitalized during the first 18 days of life for feeding difficulties and was fed later by gastrostomy. The patient had delayed milestones and psychomotor regression. She sat assisted at 2 years 4 months. At presentation, we noted dysmorphic features (small mouth, flat nasal bridge, long palpebral fissure, hypertelorism, low-set ears, high-arched palate, clasped thumb, clinodactyly), very dry skin (pachylosis), corneal ulceration and opacity, no eye contact, microcephaly, generalized hypotonia (Figure 2). She had a positional spinal deformity that did not require spinal bracing. Neither contractures nor hip dysplasia were observed.

The patient benefited from intensive weekly physical therapy (three times per week), occupational and speech therapies. She developed epileptic spasms at 2 months, then generalized seizures provoked by infection or fever. Upon the last examination at the age of 6.5 years, she had poor head control, never spoke, never held eye contact or engaged in social interactions. The patient passed away at 6.5 years due to respiratory insufficiency triggered by an infection. An autopsy was not performed.

Work-up showed ventricular septal defect, unilateral hearing loss, cortical blindness (rod abnormality on electroretinogram, normal visual evoked potentials and funduscopic exams). Brain MRI showed white matter abnormalities, cortical and bilateral subcortical atrophy in the frontotemporal regions, prominent perivascular spaces with surrounding gliosis in periarterial white matter. No calcifications were seen on the brain CT scan. EEG performed upon hospitalization for a generalized tonic–clonic seizure at 9 months, showed a diffuse slowing of the background activity and abnormal spikes and spike-waves. First-line biological investigations showed elevated blood concentrations of transaminases (ASAT = 84 UI/L (N < 35; ALAT = 31 UI/L (N < 35)). An extensive etiological exploration including genetic (karyotype and CGH array showed pericentric inversion of the short arm of chromosome 8; new generation sequencing panel for lysosomal storage disorders), and metabolic investigations (amino acids, organic acids, acylcarnitines, lysosomal enzymes) showed no underlying abnormalities consistent with the clinical phenotype. Given the complex clinical picture and the multisystemic involvement, trio-based exome sequencing was conducted. Informed and written consent was obtained from both parents for genetic analyses and picture release. 

### 2.2. Whole Exome Sequencing

DNA was isolated from fresh whole blood of both unaffected parents and the proband using a Flexigene DNA kit (Qiagen). Exome sequencing was performed in the IRIB-Rouen University Hospital facility (Service Commun de Génomique). Briefly, library preparations were obtained using Agilent Sureselect all exons human V7 kit and 75-bp paired-end sequencing was performed on an Illumina NextSeq 500 sequencer with a target mean depth of sequencing of 100× [19]. Bioinformatics pipeline was performed as previously described [20]. Briefly, single nucleotide variants and indels were detected using a pipeline based on BWA 0.7.17, Picard 2.18.0 and GATK 4.0.6.0 following best practices and CNVs were called using a CANOES-based pipeline [21]. SNVs/indels were annotated using SNPEff and SNPSIFT, including in-house annotations. Variant interpretation focused on de novo variants, rare (allele frequency below 1% in the gnomAD database) homozygous or compound heterozygous variants and all rare coding and intronic variants in flanking exonic regions falling into genes associated with Mendelian disorders based on OMIM. Variants of interest were confirmed by Sanger sequencing.

### 2.3. Urine Oligosaccharide Screening

Urine oligosaccharides were analyzed by matrix-assisted laser desorption-time of flight (MALDI-TOF) mass spectrometry at Mayo Clinic, Biochemical Genetic Laboratory, modified from a previously published method [22]. This method allows the identification of oligosaccharides with an m/z ranging from 585 to 4000. Urine oligosaccharide profiles of patients with NGLY1 deficiency show an increased excretion of n oligosaccharide with m/z = 990 corresponding to Neu5Ac1Hex1GlcNAc1-Asn. This component has been previously identified in aspartylglucosaminuria (OMIM #208400) patients [23], however, the full oligosaccharide profiles are different in these two conditions.

### 2.4. Literature Review

A literature review, up to September 2020, was performed using the terms “NGLY1-CDDG”, “NGLY1” and “NGLY1 deficiency” using Pubmed.

## 3. Results

### 3.1. Molecular and Biochemical Findings

Trio-based exome sequencing revealed one single variant of interest: a homozygous novel missense variant NM_018297.3: c.982C>T; p.(Arg328Cys) in the *NGLY1* gene. This variant was present in a heterozygous state in both unaffected parents. This finding prompted us to search for NGLY1 deficiency. Specific biochemical investigation of urinary oligosaccharides evidenced an abnormal species with m/z = 990.5 corresponding to Neu5Ac1Hex1GlcNAc1-Asn (Supplementary Figure S1). This result is consistent with a diagnosis of NGLY1 deficiency. 

Taken together, we considered that this NGLY1 variant can be rated as likely pathogenic (class 4 of the ACMG-AMP recommendations [24].

### 3.2. Literature Review

Our literature search identified 36 patients with pathogenic variants in NGLY1. The molecular and clinical overviews of reported cases [1,3,4,5,6,7,8,9,10,11,23,25,26,27,28] are summarized in Figure 1B and Figure 2A and detailed in the Supplementary Table S1. The most frequently associated clinical signs are psychomotor retardation (36/36), abnormal movements (30/30), hypolacrima or alacrima (30/30) and cytolysis (30/30).

## 4. Discussion

NGLY1 deficiency is a newly recognized condition with a multisystemic phenotype.

We describe here a female patient with a complex multisystemic involvement. She presented three out of four symptoms of the classical tetrad seen in these patients and that includes developmental delay, hypolacrima, transiently elevated transaminases, and hyperkinetic movement disorders [1,7,9,29]. Neither documented movement disorders in our patient nor orthopedic complications were observed. Orthopedic complications are usually observed later in the disease progression and our patient was on extensive physical therapy which might have delayed the occurrence of this feature [29]. Further neurological complications were also noted including psychomotor regression, microcephaly, hypotonia and seizures as in the majority of cases with NGLY1-CDDG [1,4,5,7,8,9,10,11,25,26,27].

After extensive investigations, trio-based whole-exome sequencing was conducted and revealed a homozygous novel missense variant NM_018297.3:c.982C>T, p.(Arg328Cys) in the NGLY1 gene. This variant was predicted to be deleterious by the following in silico algorithms, SIFT, PolyPhen2, MutationTaster and M-CAP. Furthermore, this variant affects the same codon as another pathogenic variant, c.982C>G; p.(Arg328Gly), recently described in a patient with NGLY1 deficiency [4]. Structural studies demonstrated that the latter variant may alter amino acid covalent binding. Besides, the mutated protein showed a defective enzyme activity [4]. Thus, these results demonstrate that the Arg328 codon is essential for the structural integrity and the catalytic activity of NGLY1. These findings emphasize the potential pathogenic effect of the variant identified in our patient. Besides, NGLY1 deficiency was confirmed upon the identification of an abnormal urine oligosaccharide (Neu5Ac1Hex1GlcNAc1-Asn). This component is identified in NGLY1-CDDG and aspartylglucosaminuria patients, but in the latter condition other additional characteristic high-mass species are present [23].

The literature review retrieved 36 NGLY1-CDDG patients with genetic confirmation [1,3,4,5,6,7,8,9,10,11,23,25,26,27,28]. The most prevalent clinical features are presented in Figure 1B. All patients have developmental delays. Seizures of different types that could be easily controlled by antiepileptic drugs or as part of refractory epilepsy have been described [1]. Hyperkinetic movement disorders include choreiform, athetoid, dystonic, myoclonic, tremor, dyskinetic, and dysmetric movements [1,7,8,9,10,26]. An increase in blood transaminase concentration was often present in the first two years of life along with feeding difficulties. Ophthalmological complications consisted essentially of hypolacrima/alacrimia but also lagophthalmos, ptosis, exotropia/esotropia, retinal pigmentary changes, corneal disease and refractive errors [1,4]. Concerning hearing, transmission abnormalities due to poor myelination were rarely seen [1]. Growth retardation could be observed even prenatally with documented intrauterine growth retardation and some patients were born prematurely [4,7,8,10,25]. Some of these patients presented associated neuromuscular dysfunction (axonal or demyelinating peripheral neuropathy, small fiber neuropathy, scoliosis) and orthopedic complications (joint contractures, fractures, hip dysplasia) [1,4,5,29]. At the molecular level, 32 different variants, including our case, were reported in the *NGLY1* gene (Figure 1B, Supplementary Table S1). Pathogenic variants were mostly truncating variants introducing premature termination codon (PTC) through nonsense or splice SNVs or frameshift indels throughout the gene sequences. The most recurrent variant, c.1201A>T, p.(Arg401*) accounted for 20% (15/74) of the mutant alleles and has been linked to a more severe phenotype [27]. The second most recurrent variant (9%) was also a PTC-introducing variant, through a frameshift: c.1891del, p.(Gln631Serfs*7). Nine missense variants were characterized in the *NGLY1* gene central sequences coding for the catalytic domain demonstrating that the integrity of these sequences is essential for the protein function. Here, the clinical phenotypic features were highly suggestive of NGLY1 deficiency. Indeed, in retrospect, our patient had 9 out of 10 cardinal clinical signs associated with NGLY1 deficiency as shown in Figure 2B. The biochemical validation allowed us to confirm the diagnosis and hence provide genetic counseling in this family.

## 5. Conclusions

In summary, this report describes the first French case of NGLY1-CDDG. Most cases described in the literature were diagnosed by molecular analysis using whole exome or genome sequencing as in our case. Very few cases have been diagnosed through the identification of specific biochemical biomarkers [10,27]. Clinical and biological knowledge of this pathology greatly improved over the last few years. Henceforth, the association of key clinical signs should prompt to evoke NGLY1 deficiency, to carry out specific biochemical and molecular explorations thus, avoid diagnostic wandering and maybe allow therapy in the incoming years.

## Data Availability

All data that support the findings are included in the manuscript and in the supplemental data.

## Supplementary Materials

The following are available online at https://www.mdpi.com/2075-1729/11/3/187/s1, Figure S1: Urine oligosaccharide profiling by MALDI-TOF for the identification of NGLY1 deficiency. Table S1: Clinical and molecular metadata.
