# Supplementary material for: NGLY1 Deficiency: A Rare Newly Described Condition with a Typical Presentation
Source: Life (Basel). 2021 Feb 27;11(3):187. doi: 10.3390/life11030187 (PMC7996810; doi:10.3390/life11030187)
Supplement: Supplementary file 1 [file life-11-00187-s001.zip › suppl fig-for XML.docx]

Supplementary Materials of NGLY1 Deficiency: A Rare Newly Described Condition with a Typical Presentation

**Figure S1.** Urine oligosaccharide profiling by MALDI-TOF for the identification of NGLY1 deficiency. MS/MS product ion scans of Neu5Ac1Hex1GlcNAc1-Asn in a positive control with NGLY1 deficiency and our patient. (**A**) positive control. (**B**) Our patient. We note an abnormal species with m/z = 990.5 corresponding to Neu5Ac1Hex1GlcNAc1-Asn oligosaccharide.
